# Expression of Chemokine Receptors on Peripheral Blood T Cells in Children with Chronic Kidney Disease

**DOI:** 10.1155/2015/536894

**Published:** 2015-03-18

**Authors:** Maria Szczepańska, Łukasz Sędek, Irena Makulska, Krystyna Szprynger, Bogdan Mazur, Joanna Bulsa, Danuta Zwolińska, Jacek Karpe, Katarzyna Ziora, Tomasz Szczepański

**Affiliations:** ^1^Dialysis Division for Children, Department and Clinic of Pediatrics, SMDZ in Zabrze, SUM in Katowice, Ulica 3 Maja 13/15, 41-800 Zabrze, Poland; ^2^Department of Pediatric Hematology and Oncology, SMDZ in Zabrze, SUM in Katowice, Ulica 3 Maja 13/15, 41-800 Zabrze, Poland; ^3^Department of Pediatric Nephrology, Wrocław Medical University, Ulica Borowska 213, 50-556 Wrocław, Poland; ^4^Department of Microbiology and Immunology, SMDZ in Zabrze, SUM in Katowice, Ulica Jordana 19, 41-808 Zabrze, Poland; ^5^Department of Anesthesiology and Intensive Care, SMDZ in Zabrze, SUM in Katowice, Ulica 3 Maja 13/15, 41-800 Zabrze, Poland

## Abstract

Chemokine receptors play a role in leukocyte recruitment, activation, and maintaining effector functions and regulate adaptive immune response and angiogenesis. The study aimed at flow cytometric analysis of T cell subsets with selected surface chemokine receptors (CCR4, CCR5, CCR7, CXCR3, and CXCR4) or receptor combination in peripheral blood of children with chronic kidney disease (CKD) on hemodialysis (HD). The percentage of T lymphocytes with CD8 and combined CD28,CCR7 expression was higher in HD children. The percentage of T lymphocytes expressing CCR7, CD28,CCR7, and CXCR4,CD8 was increased in children on conservative treatment. Total number (tn) of CXCR4+ cells was reduced in children on hemodialysis. The tn of T CXCR3+ cells was lower in children on conservative treatment. During HD the percentage of T CD4+ cells was higher and of T CXCR3+ lymphocytes was lower after HD session as compared to 15 min of session duration. During HD tn of T cells with expression of CCR4, CCR5, CCR7, CXCR3, and CXCR4 was constant. The alteration of chemokine receptors expression in children with CKD occurs early in the development. Diminished expression of CXCR3, CXCR4 on T cells in patients with CKD on HD might result in impaired inflammatory response. Increased CCR7+ T cell percentage could be responsible for the alteration of migration of cells into secondary lymphatic organs.

## 1. Introduction 

It is well-established that, after the initial bacterial, fungal, or viral infection, further destructive processes in surrounding tissues are the result of altered host immune-inflammatory response. Chemokine receptors play a role in leukocyte recruitment, activation, and maintaining effector functions of immunocompetent cells, and they regulate adaptive immune response and angiogenesis through the interactions with adhesion molecules and cytokines [1]. They are classified as CCR, XCR, CXCR, or CX3CR like equivalent four classes of chemokines. Additionally they are numbered with regard to the order of identification [2, 3].

During past decades several groups tried to highlight the role of chemokines and chemokines receptors system in kidney disease with particular interest in chronic kidney disease (CKD) [4, 5].

Interventional studies in rodents documented the presence of CCR2 receptor on monocytes infiltrating kidney interstitial area, which were involved in experimental kidney inflammation [6]. Furuichi et al. pointed out that, among the pairs chemokines/chemokine receptors, CCR2-mediated macrophage infiltration has affected tubular necrosis after ischemic acute kidney injury and IFN-*γ*-inducible protein 10—producing macrophages which participated in the processes of tubular epithelial cells regeneration. Additionally CX3CR1-mediated macrophages and platelets were involved in interstitial fibrosis in CKD [1]. Segerer at al. demonstrated the infiltrate expressing CXCR3 in the tubulointerstitium in renal biopsies of patients with lupus nephritis with a correlation between the number of infiltrating CXCR3+ T cells and the degree of kidney dysfunction [7].

The application of chemokine receptors antagonists into clinical practice might have beneficial effect on CKD progression, which needs further confirmation in clinical studies [8].

Borkar et al. highlighted the importance of the CCR and CX3CR1 genotyping in relation to progression to end-stage renal disease. They confirmed that early genotyping of CCR5 G59029A and CX3CR1 T280M and V249I polymorphism help to identify the subjects with disease progression in advanced CKD cases among the population of North Indians [9].

Chu and coworkers documented that CXCR4 inhibition blunted the accumulation of T-lymphocytes in the heart and kidneys so this receptor could be involved in the pathogenesis of hypertension, inflammation, and fibrosis induced by mineralocorticoid excess [10]. Yuan et al. in experimental study demonstrated that following unilateral ureteral obstruction gene and protein expression of CXCR4 was simultaneously significantly upregulated in nephron tubular cells. The increased tubular CXCR4 expression correlated with increased cells dedifferentiated state leading to an increased mRNA expression of PDGF*α* and TGF*β*1 and loss of BMP7 [11].

Children with CKD similar to adult population show the features of altered cellular immunity. Only limited data are available on different receptor expression on peripheral blood (PB) T cell subsets in children with CKD on renal replacement therapy [12, 13]. All mentioned below surface receptors were chosen as having a potential involvement in the process of chronic kidney disease.

## 2. Objectives

The study aimed at multiparameter analysis of the absolute numbers and percentages of T cell subsets with selected chemokine receptors (CCR4, CCR5, CCR7, CXCR3, and CXCR4) or scheduled receptor combination in children with CKD on hemodialysis.

## 3. Material and Methods

The study group consisted of 12 children and young adults with CKD on hemodialysis and 15 children on conservative therapy. Gender, mean age, anthropometric data, blood pressure values, and laboratory tests values are shown in Table 1. Two predialysis children (13,3%) were classified as stage 5, 4 (26,7%) as stage 4, 6 (40%) as stage 3, and 3 (20%) as CKD stage 2. Forty-one healthy individuals served as a control group. During the study children were in stable clinical condition without the features of acute infection. All the children remained on pharmacotherapy of CKD (diuretics, renoprotection (enalapril and sartan), carbohydrate supplementation, antihypertensive agents, and ferric and folate formulas). Twenty-one children received erythropoietin.

Dialysis was performed using Fresenius 2008 C and A (Fresenius Medical Care AG, Bad Homburg, Germany) and Dialog machines (B. Braun AG, Melsungen, Germany). Bicarbonate buffered dialysis fluid was applied. Water for hemodialysis was prepared by reverse osmosis and bacteriologically tested according to European standards. The mean time of session was 4 hours (3,5–5,0 hours). The velocity of dialysate flow was 500 mL per minute, and blood flow 150–250 mL per minute. Low molecular weight heparin was used for anticoagulation during hemodialysis session.

The expression of surface antigens was evaluated on PB mononuclear cells using multicolor flow cytometry in FACSCanto II cytometer (Becton-Dickinson, Biosciences, San Jose, CA, USA). The panel of monoclonal antibodies for lymphocyte subpopulations flow cytometric examination is described in Table 2. The sample of 3 mL of heparinized peripheral blood was drawn for each evaluation. For flow cytometric examination the method of whole blood staining of the respective cell surface molecules with subsequent erythrocytes lysis was applied. The cells were incubated with six directly labeled monoclonal antibodies in each tube. After the end of incubation erythrocytes were lysed by FACS Lysing Solution (Becton-Dickinson Biosciences, San Jose, CA, USA). After rinsing in phosphate-buffered saline (PBS) including NaCl, Na_2_HPO_4_, NaH_2_PO_4_, and NaN_3_, pH (25°C) 7.2 ± 0.1, the cells were measured in flow cytometer. The lymphocyte population was gated on the basis of forward–sideward scatter. Data were registered and analyzed by the Diva (Becton-Dickinson, Immunocytometry Systems, San Jose, CA, USA).

Absolute and relative values of particular subsets were correlated with age, anthropometrical parameters, creatinine, BUN, hemoglobin, adequacy markers, and mean arterial pressure (MAP). Systolic and diastolic blood pressure (BP sys and BP dia) were measured before a dialysis session in HDs or during an outpatient clinic appointment in patients on conservative treatment and in control group. MAP was defined as BP dia + (BP sys-BP dia)/3.

Absolute and relative values of particular subsets were correlated with age, anthropometrical parameters (weight, height, and BMI), creatinine, BUN, hemoglobin, hematocrit, erythrocyte count, adequacy markers, dialysis treatment duration, CKD duration, and mean arterial pressure (MAP).

In the statistical analysis we used the ANOVA test. Correlations between T cell subpopulation values and other parameters were analyzed with Pearson's test. The *P* values of <0.05 were considered significant.

## 4. Results

Tables 3 and 4 describe the percentage and absolute values of T cell subpopulations in children with CKD and healthy controls. We have demonstrated that the percentage of T lymphocytes with the surface expression of CD8 and combined CD28,CCR7 in peripheral blood in children on HD was higher as compared to healthy controls. The percentage of T lymphocyte subpopulations expressing CCR7, CD28,CCR7 and CXCR4,CD8 was also increased in children on conservative treatment comparing to control group.

The total number of T cells with CXCR4 surface antigen was significantly reduced in children on hemodialysis as compared to healthy children. The total number of T cells with CXCR3 surface antigen was significantly lower in children with CKD on conservative treatment as compared to healthy children.

During hemodialysis session the percentage of CD4+ T cells was higher after the session as compared to the beginning and the percentage of CXCR3 expressing T lymphocytes was lower after the HD session as compared to measurement at 15 min after beginning of the session (Table 5).

During hemodialysis session the absolute number of all T cells with expression of single and/or combination of examined chemokine receptors (CCR4, CCR5, CCR7, CXCR3, and CXCR4) was constant (Table 6).

We showed statistically significant correlation of erythrocyte count with the percentage of CD3+CXCR3+CXCR4+ lymphocytes, MAP with the absolute number of CD3+CCR5+CXCR3+ lymphocytes, and KT/V with the absolute number of CD3+CCR7+ and CD3+CD45RO+CCR7+ lymphocytes (Table 7).

## 5. Discussion

Chemokine receptor blockade might selectively protect from the influx of proinflammatory leukocytes with no influence on white cells with immunoregulatory or anti-inflammatory character. Concerning kidney disease, the role of chemokines in interactions between cytokine action, vasoactive substances, and corresponding target cells has been emphasized in the progression of chronic interstitial fibrosis [4, 8].

CXCR3 is the unique receptor for three chemokines representing the CXC chemokine family, that is, CXCL9 (monokine induced by interferon-*γ* (IFN-*γ*), MIG), CXCL10 (IFN-*γ*-inducible protein 10, IP-10), and CXCL11 (IFN-*γ*-inducible T cell *α* chemoattractant, I-TAC). CXCR3 pathway is involved in the development of autoimmune diseases. This chemokine receptor plays a role in creating local amplification loops of inflammation in target organs, thereby inducing worsening of clinical appearance especially in systemic lupus erythematosus and rheumatoid arthritis [14, 15]. The activation of CXCR3 also stimulates Th1-type dependent cytokine production with synchronized downregulation of Th2 cytokines. It also plays a role in regulation of tumor growth and metastasis as well as in mechanisms of tissue repair and wound healing.

Chemokine receptors CXCR3 and CCR4 present on the surface of T lymphocytes could be regarded also as the markers of early lymphocyte T CD4+ maturation before their differentiation into T_EM_ and T_CM_ cells. In our study the absolute number of CXCR3+ T cells was reduced in examined children with CKD on conservative treatment and in HD patients. As CXCR3 is induced by an inflammatory status, regulating T cell activation we can conclude this phenomenon as impaired inflammatory response. No change was observed in Th1 lymphocyte subpopulation with surface CXCR3 receptor and Th2 lymphocyte with CCR4 in children on hemodialysis in comparison to controls. The percentage of CXCR3+ T lymphocytes was decreased after the HD session in comparison to the burst induced in the first quarter of session with no difference from baseline value. This phenomenon was not described in pediatric literature so far. It suggests that uremia has an influence of this stage of lymphocyte development but hemodialysis treatment could restore that alteration. CXCR3 expression has been proposed as a marker for T memory response, as viral recall responses for T CD8+ and CD4+ cells are largely restricted to CXCR3+ cells [16]. Impaired answer for vaccination in CKD could be also the sequel of T CXCR3+ cells depletion. Elevated blood pressure could have a negative influence on CXCR3+ T cell numbers in children with CKD. Chiesa et al. described decreased polarization into Th1 and Th2 cells in 4 from 6 children with CKD after one year of peritoneal dialysis treatment. However, these authors evaluated the production of IFN*γ* and IL-4 by T lymphocytes not the pattern of chemokine receptors [17].

It was documented that patients with high serum level of CXCL10 before kidney transplantation are more prone to acute Th1-mediated rejection. Long-term administration of neutralizing anti-CXCR3 antibodies prevents alloreactive T cell-mediated graft-versus-host disease in a mouse model [18].

Lymphocytes Th1 also show the expression of chemokine receptor CCR5. In our previous report we demonstrated the high CCR5 expression in the youngest group of healthy children, which could be responsible for the alteration of cellular immune response in that age group [19]. There was no difference in CCR5 percentage and/or absolute value of this chemokine receptor in children with CKD regardless of treatment modality: conservative or hemodialysis.

Sherry et al. did not describe any changes in CCR5 receptor expression on monocytes in children on hemodialysis. However, these authors did not evaluate the expression of this receptor on lymphocytes. Interactions of chemokine CCR5 receptor with specific ligands, MIP-1*α*, MIP-1*β*, (CCL3/CCL4), or RANTES (CCL5), release signaling cascade inducing migration of immunocompetent cells to the site of inflammation [20]. In the literature the important role of CCR5 was described in rheumatoid arthritis, multiple sclerosis, transplant rejection, diabetes, multiple myeloma, and gastrointestinal diseases. CCR5 enables intracellular migration of HIV-1 [15]. It was documented that patients with type 2 diabetes present overexpression of the CCR5 receptor on peripheral blood mononuclear cells, which should be additionally considered as a factor that promotes the development of atherosclerosis in diabetic patients [21].

Szalai et al. observed that patients with atherosclerosis with CCR5 gene mutation, deletion 32 (CCR5Δ32), have better outcome, which is associated with hypofunction of the receptor. CCR5Δ32 genotype has also better prognosis of longer survival in type 2 diabetes [22].

As the price of genetic testing is rather high Muntinghe et al. evaluated the potential cost-effectiveness of pharmacologically blocking the CCR5 receptor in inflamed dialysis patient with the CCR5 insertion/insertion genotype and found it to be similar to the cost of existing treatment modalities for dialysis patients [23]. The role of CCR5 and CXCR4 chemokine receptors in allergic processes occurring in lungs (fibrosis) was described [9]. The study of Bot et al. documented that blockade of the CXCL12 chemokine (SDF-1*α*)/CXCR4 axis on leukocytes induces atherosclerotic plaque progression in mice, which may be associated with an increased adherence to plaque endothelium and progression of the disease [24]. SDF-1/CXCR4 pathway plays an important role in neoplasia and in metastases formation as CXCR4 is most abundant expressed on cancer cells. Increased expression of CXCR4 chemokine receptor has also prognostic value in* Rhabdomyosarcoma *[25]. New therapeutics directed anti-CXCR4 can block the SDF-1/CXCR4 interaction or inhibit downstream intracellular signaling, which represents a significant antitumor option [26]. As CXCR4 is also a coreceptor for HIV T-lymphotropic virus, enabling its intracellular penetration, agents blocking the receptor are important in the treatment of AIDS [27]. In our study the expression of CXCR4 receptor on T lymphocytes in children on HD was significantly reduced, but the concurrent determination of CD4 and CD8 expression failed to confirm this data. It could be explained by only partial coexpression of CXCR4 surface molecule with other antigens and by the low number of examined children, which is often the weak point of pediatric studies.

On the basis of biopsy specimen examination literature data show that the blockade of proinflammatory cells bearing on their surface the CCR5 chemokine receptor allows the inhibition of inflammatory processes and reverse transplant rejection [18].

Surface molecule CCR7 is a homing receptor directing the migration of cells into secondary lymphoid organs and CCR7-mediated signals contribute to other immune processes such as modulation of cell proliferation and activation or differentiation of various cellular subpopulations [28, 29].

Recent results demonstrate that the CCR7-dependent contacts of T cells and dendritic cells are also essential for the induction of peripheral tolerance and the regulation of the immune response by CD4+CD25+ regulatory T (TReg) cells. Although CCR7 has been identified as a lymph-node homing receptor, there is accumulating evidence that this receptor is also involved in lymphocyte recirculation [30]. Our data have shown that percentage of T cells with surface antigen CCR7 and combination of antigens CCR7, CD28 was increased in children with CKD on conservative treatment. CD28 is a costimulatory molecule, so its preservation enables antibodies production and could play a role in immunotolerance. On the other hand Schaeuble et al. found that long-term activation of isolated human T cells through CD3/CD28 attenuated CCR7-driven chemotaxis, whereas short-term action significantly enhanced CCR7-mediated (not CXCR4-mediated) migration efficiency. Short-term activation of T cell receptor particularly enhanced the migratory response of naive T cells of CD4 and CD8 subpopulations [31]. Moschovakis et al. confirmed that the absence of CCR7 signaling favored polarization towards Th2 cells, dislocation of T helper cells into the B-cell follicles, and, as a consequence, the activation of B-cells [28].

## 6. Conclusions

The alteration of chemokine receptor expression in patients with CKD occurs at the early stages of the development also in pediatric population.

Diminished expression of CXCR3, CXCR4 chemokine receptors on T cells in patients with CKD on hemodialysis might result in impaired inflammatory response. Increased CCR7+ T cell percentage could be responsible for the alteration of migration of cells into secondary lymphatic organs.

## Figures and Tables

**Table 1:** Age, gender, anthropometric parameters, selected laboratory tests results, and dialysis duration in studied children.

Analyzed parameter	HD group (*n* = 12)	CKD group (*n* = 15)	Control group (*n* = 41)	*P* ANOVA
1	2	4	5	6
Age [years]	9,6–24,6	4,6–17,7	6,4–19,6	
Mean age [years]	16,5 ± 4,5^a,c^	13,0 ± 4,6	12,5 ± 4,1	**0,0001**
Gender [F/M]	5/7	5/10	16/25	
Dialysis duration [years]	3,4 ± 3,5			
CKD duration [years]	7,8 ± 5,8	6,6 ± 5,2		
Weight [kg]	42,0 ± 7,9	41,7 ± 17,9	46,7 ± 17,8	NS
Height [cm]	148,0 ± 11,6	146,3 ± 30,1	157,3 ± 23,6	NS
BMI [kg/m^2^]	19,2 ± 3,6	18,2 ± 2,3	18,9 ± 3,5	NS
MAP [mmHg]	96,7 ± 10,8^a,c^	79,0 ± 10,2	76,7 ± 13,6	**0,0035**
Hemoglobin [g/dL]	9,1 ± 1,2^a,c^	12,2 ± 1,9^b^	13,6 ± 1,4	**0,0000**
Hematocrit [%]	26,5 ± 3,1^a,c^	35,0 ± 3,9^b^	39,5 ± 4,7	**0,0000**
Erythrocytes [1 ∗ 10^12^/L]	3,0 ± 0,5	4,2 ± 0,6	4,7 ± 0,4	NS
Thrombocytes [1 ∗ 10^9^/L]	234,9 ± 119,0^c^	253,1 ± 135,2^b^	308,5 ± 89,6	**0,0020**
Leukocytosis [1 ∗ 10^9^/L]	5,7 ± 1,6^c^	5,9 ± 1,8^b^	7,7 ± 1,9	**0,0005**
Creatinine [*μ*mol/L]	960,3 ± 270,0^a,c^	282,5 ± 179,7^b^	66,5 ± 16,9	**0,0000**
Urea nitrogen [mmol/L]	24,0 ± 6,1^a,c^	14,7 ± 7,3^b^	4,3 ± 1,21	**0,0008**
GFR [mL/min]		34,4 ± 20,2^b^	124,2 ± 23,2	**0,0000**
Kt/V	1,1 ± 0,3			
URR [%]	58,6 ± 10,5			

Absolute values are shown as mean ± standard deviation.

*P*: significance in ANOVA test.

*P* < 0,05, ^a^CKD versus HD, ^b^CKD versus control group, and ^c^HD versus control group.

**Table 2:** The panel of monoclonal antibodies for lymphocyte subpopulations flow cytometric examination.

Number	FITC	PE	PerCP	PE-Cy7	APC	APC-Cy7
1	CD4 BD	**—**	CD3 BD	CCR4 BD Pharmingen	**—**	CD8 BD

2	CD45RO Dako	CD197 = CCR7 BD Pharmingen	CD3 BD	CD19 BD	CD28 BD Pharmingen	CD8 BD

3	CD4 BD	CD183 = CXCR3 BD Pharmingen	CD3 BD	CD195 = CCR5 BD Pharmingen	CD184 = CXCR4 BD Pharmingen	CD8 BD

BD: Becton Dickinson, San Jose, CA, USA.

BD Pharmingen: Becton Dickinson Pharmingen-Biosciences, La Jolla, CA, USA.

Dako: Dakopatts, Glostrup, Denmark.

FITC: fluorescein isothiocyanate; PE: phycoerythrin; PerCP: peridinin-chlorophyll; PE-Cy7: phycoerythrin-cyanin 7; APC: allophycocyanin; APC-Cy7 allophycocyanin-cyanin 7.

**Table 3:** Percentage values of T cell subpopulations, including populations with expression of receptors for selected chemokines in examined children and healthy controls.

Lymphocyte subpopulations [%]	HD group	CKD group	Control group	*P*
T lymphocytes	71,0 (53,4–90,1)	69,9 (49,8–89,9)	69,3 (36,7–91,9)	0,2903
CD4+ T cells	41,8 (15,2–55,5)	36,6 (20–54,8)	34,2 (11,4–58,0)	0,3171
CD8+ T cells	31,2 (13,5–45,0)	30,7 (15,5–45,4)	22,2 (8,4–42,1)	**0,0001**
CCR7+ T cells	15,4 (2,1–33,7)	18,1 (5,3–30,1)	2,4 (0,3–49,6)	**0,0052**
CD45RO+CCR7+	2,4 (0,4–10,1)	3,1 (0,6–21,2)	0,9 (0,3–21,2)	0,3274
CD28+CCR7+ T cells	15,1 (2,1–32,4)	16,5 (4,9–29,5)	2,6 (0,3–48,5)	**0,0412**
CXCR4+ T cells	10,6 (1,3–45,9)	23,1 (4,4–45,5)	14,4 (2,9–60,1)	0,1030
CXCR4+CD4+ T cells	2,6 (0,001–31,7)	6,5 (0,3–24,3)	5,5 (0,0004–38,7)	0,4996
CXCR4+CD8+ T cells	6,7 (1,3–21,3)	15,0 (3,1–29,1)	7,0 (1,1–32,0)	**0,0233**
CXCR4+CCR5+ T cells	0,8 (0,001–8,5)	2,5 (0,6–17,9)	2,5 (0,0002–26,0)	0,0795
CCR5+ T cells	13,0 (3,6–26,9)	15,1 (5,1–30,3)	12,8 (2,2–53,0)	0,4015
CCR5+CD4+ T cells	3,2 (1,3–5,6)	3,4 (1,4–5,6)	3,4 (0,6–9,5)	0,6438
CCR5+CD8+ T cells	7,4 (1,7–21,7)	9,3 (2,6–18,7)	5,8 (1,1–41,3)	0,9799
CCR4+ T cells	5,0 (0,3–22,3)	6,0 (0,8–33,6)	7,5 (1,0–31,6)	0,6464
CCR4+CD4+ T cells	4,4 (0,9–47,3)	4,5 (1,7–12,3)	5,7 (0–15,9)	0,2978
CCR4+CD8+ T cells	2,0 (0,002–14,2)	1,1 (0,001–34,3)	0,9 (0,0002–24,7)	0,7969
CXCR3+ T cells	31,8 (14,8–42,7)	30,6 (18,7–43,3)	23,4 (11,3–48,8)	0,9798
CXCR3+CD4+ T cells	10,7 (4,9–18,1)	10,0 (4,5–19,3)	9,3 (1,7–17,0)	0,5450
CXCR3+CD8+ T cells	14,4 (8,5–33,2)	17,1 (8,9–26,7)	16,5 (7,2–27,8)	0,8588
CXCR3+CCR5+ T cells	10,6 (3,1–23,4)	11,8 (4,1–26,6)	6,9 (1,3–26,9)	0,2822
CXCR3+CXCR4+ T cells	7,4 (0,7–20,3)	14,3 (2,1–26,9)	5,9 (1,0–15,9)	0,1994

Percentage values are shown as median value (minimum–maximum).

*P*: significance in ANOVA test.

**Table 4:** Absolute values of T cell subpopulations, including populations with expression of receptors for selected chemokines in examined children and healthy controls.

Lymphocyte subpopulations [G/l]	HD group	CKD group	Control group	*P*
T lymphocytes	1.4 ± 0.5	1.5 ± 0.9	1.8 ± 0.6	0,0577
CD4+ T cells	0.78 ± 0,4	0.85 ± 0.6	0.94 ± 0.4	0,1629
CD8+ T cells	0.58 ± 0.3	0.65 ± 0.3	0.62 ± 0.3	0,7768
CCR7+ T cells	0.31 (0.04–0.8)	0.41 (0.1–0.7)	0.06 (0.005–1.8)	0,3883
CD45RO+CCR7+	0.04 (0.011–0.1)	0.06 (0.01–0.35)	0.02 (0.01–0.58)	0,8162
CD28+CCR7+ T cells	0.31 (0.04–0.8)	0.38 (0.1–0.7)	0.06 (0.01–1.8)	0,7937
CXCR4+ T cells	0,27 ± 0,21	0,49 ± 0,32	0,55 ± 0,46	**0,0387**
CXCR4+CD4+ T cells	0,04 (0,00003–0,4)	0,013 (0,02–0,4)	0,2 (0,00001–0,9)	0,0621
CXCR4+CD8+ T cells	0,1 (0,03–0,6)	0,3 (0,04–0,9)	0,2 (0,02–1,1)	0,3762
CXCR4+CCR5+ T cells	0,01 (0,00002–0,2)	0,1 (0,02–0,4)	0,1 (0,00001–0,2)	0,0639
CCR5+ T cells	0,32 ± 0,22	0,29 ± 14	0,41 ± 0,32	0,2499
CCR5+CD4+ T cells	0,07 ± 0,04	0,07 ± 0,03	0,1 ± 0,07	0,0636
CCR5+CD8+ T cells	0,1 (0,02–0,6)	0,2 (0,05–0,4)	0,1 (0,02–0,6)	0,4947
CCR4+ T cells	0,16 ± 0,13	0,16 ± 0,15	0,27 ± 0,26	0,1433
CCR4+CD4+ T cells	0,1 (0,02–1,2)	0,1 (0,03–0,2)	0,1 (0–0,4)	0,3578
CCR4+CD8+ T cells	0,05 (0,00004–0,3)	0,02 (0,00002–0,8)	0,03 (0,00001–0,9)	0,9535
CXCR3+ T cells	0,63 ± 0,31	0,63 ± 0,26	0,84 ± 0,33	**0,0294**
CXCR3+CD4+ T cells	0,2 ± 0,09	0,2 ± 0,07	0,25 ± 0,12	0,1813
CXCR3+CD8+ T cells	0,35 ± 0,22	0,35 ± 0,15	0,46 ± 0,21	0,0789
CXCR3+CCR5+ T cells	0,25 ± 0,17	0,23 ± 0,12	0,30 ± 0,23	0,5108
CXCR3+CXCR4+ T cells	0,17 ± 0,14	0,26 ± 0,18	0,25 ± 0,23	0,4623

Absolute values are shown as mean ± standard deviation for Gaussian distribution, otherwise as median (minimum–maximum).

*P*: significance in ANOVA test.

**Table 5:** Percentage values of T cell subpopulations, including populations with expression of receptors for selected chemokines in children on maintenance hemodialysis.

Lymphocyte subpopulations [%]	Before HD	HD 15 min	After HD	*P*
T lymphocytes	71,0 (53,4–90,1)	73,3 (64,3–99,3)	74,5 (30,4–90,1)	0,4431
CD4+ T cells	41,8 (15,2–55,5)	39,1 (8,4–60,1)	46,7 (31,0–66,2)	**0,0211**
CD8+ T cells	31,2 (13,5–45,0)	21,2 (10,7–44,0)	25,4 (13,1–76,5)	0,3521
CCR7+ T cells	15,4 (2,1–33,7)	19,4 (2,2–49,0)	14,6 (2,9–42)	0,6822
CD45RO+CCR7+	2,4 (0,4–10,1)	3,2 (0,5–8.3)	2,0 (0,5–6,0)	0,6933
CD28+CCR7+ T cells	15,1 (2,1–32,4)	19,1 (2,2–48,5)	14,6 (2,4–41,5)	0,6777
CXCR4+ T cells	10,6 (1,3–45,9)	15,3 (4,7–37,8)	8,4 (1,2–42,2)	0,8561
CXCR4+ CD4+ T cells	2,6 (0,001–31,7)	6,8 (0,8–13,2)	2,1 (0,001–29,1)	0,7951
CXCR4+ CD8+ T cells	6,7 (1,3–21,3)	6,8 (2,7–34,0)	5,8 (1,2–15,0)	0,5598
CXCR4+CCR5+ T cells	0,8 (0,001–8,5)	1,1 (0,002–12,9)	0,5 (0,001–3,2)	0,3834
CCR5+ T cells	13,0 (3,6–26,9)	12,2 (4,8–32,4)	9,3 (2,4–21,1)	0,0954
CCR5+CD4+ T cells	3,2 (1,3–5,6)	3,3 (2,1–7,8)	2,4 (0,4–8,3)	0,4225
CCR5+CD8+ T cells	7,4 (1,7–21,7)	6,3 (2,1–27,2)	3,9 (1,5–15,3)	0,3314
CCR4+ T cells	5,0 (0,3–22,3)	5,2 (0,6–23,2)	7,6 (0,5–17,3)	0,9458
CCR4+ CD4+ T cells	4,4 (0,9–47,3)	4,0 (1,2–9,6)	4,1 (1,2–11,5)	0,4381
CCR4+ CD8+ T cells	2,0 (0,002–14,2)	1,5 (0,3–19,8)	1,9 (0,001–13,9)	0,9607
CXCR3+ T cells	31,8 (14,8–42,7)	35,4 (24,9–43,6)	29,5 (3,2–39,9)	**0,0294**
CXCR3+CD4+ T cells	10,7 (4,9–18,1)	12,7 (8,5–18,2)	11,5 (0,8–49,2)	0,5741
CXCR3+CD8+ T cells	14,4 (8,5–33,2)	18,5 (9,7–30,8)	14,2 (2,0–20,7)	0,1801
CXCR3+CCR5+ T cells	10,6 (3,1–23,4)	9,9 (3,7–22,4)	6,9 (1,6–22,7)	0,0892
CXCR3+CXCR4+ T cells	7,4 (0,7–20,3)	6,5 (3,1–23,7)	4,3 (0,8–18,0)	0,6788

Percentage values are shown as median value (minimum–maximum).

*P*: significance in ANOVA test.

**Table 6:** Absolute values of T cell subpopulations, including populations with expression of receptors for selected chemokines in children on maintenance hemodialysis.

Lymphocyte subpopulations [G/l]	Before HD	HD 15 min	After HD	*P*
T lymphocytes	1.4 ± 0.5	1,14 ± 0,36	1,04 ± 0,48	0,2288
CD4+ T cells	0.78 ± 0,4	0,56 ± 0,26	0,66 ± 0,25	0,6207
CD8+ T cells	0.58 ± 0.3	0,37 ± 0,24	0,46 ± 0,33	0,5184
CCR7+ T cells	0.31 (0.04–0.8)	0,32 ± 0,25	0,26 ± 0,22	0,7416
CD45RO+CCR7+	0.04 (0.011–0.1)	0,05 ± 0,04	0,04 ± 0,03	0,5355
CD28+CCR7+ T cells	0.31 (0.04–0.8)	0.30 (0.04–1.0)	0.26 (0.03–0.8)	0,7543
CXCR4+ T cells	0,27 ± 0,21	0,25 ± 0,16	0,19 ± 0,18	0,5490
CXCR4+ CD4+ T cells	0,04 (0,00003–0,4)	0.11 (0.009–0.3)	0.04 (0.00002–0.3)	0,8011
CXCR4+ CD8+ T cells	0,1 (0,03–0,6)	0.10 (0.04–0.6)	0.07 (0.02–0.2)	0,2380
CXCR4+CCR5+ T cells	0,01 (0,00002–0,2)	0,015 (0,00002–0,2)	0,008 (0,00002–0,07)	0,2148
CCR5+ T cells	0,32 ± 0,22	0,23 ± 0,14	0,15 ± 0,13	0,0666
CCR5+CD4+ T cells	0,06 (0,02–0,2)	0,06 (0,02–0,1)	0,03 (0,004–0,2)	0,4235
CCR5+CD8+ T cells	0,1 (0,02–0,6)	0.08 (0.02–0.5)	0.05 (0.02–0.3)	0,2293
CCR4+ T cells	0,16 ± 0,13	0,10 ± 0,08	0,12 ± 0,10	0,4772
CCR4+ CD4+ T cells	0,1 (0,02–1,2)	0.04 (0.02–0.1)	0.07 (0.01–0.2)	0,3097
CCR4+ CD8+ T cells	0,05 (0,00004–0,3)	0.02 (0.00004–0.3)	0.03 (0–0.3)	0,6337
CXCR3+ T cells	0,63 ± 0,31	0,55 ± 0,22	0,40 ± 0,24	0,1016
CXCR3+CD4+ T cells	0,2 ± 0,09	0,2 ± 0,08	0,2 ± 0,13	0,9143
CXCR3+CD8+ T cells	0,35 ± 0,22	0,3 ± 0,15	0,22 ± 0,1	0,1907
CXCR3+CCR5+ T cells	0,25 ± 0,17	0,19 ± 0,12	0,12 ± 0,11	0,1023
CXCR3+CXCR4+ T cells	0,17 ± 0,14	0,13 ± 0,10	0,09 ± 0,07	0,1582

Absolute values are shown as mean ± standard deviation for Gaussian distribution, otherwise as median (minimum–maximum).

*P*: significance in ANOVA test.

**Table 7:** The analysis of correlation between T lymphocyte subpopulations with chemokine receptors and other parameters in children with CKD on maintenance hemodialysis.

Parameter	Lymphocyte T subpopulation	Significance
Erythrocyte count	The percentage of CD3+CXCR3+CXCR4+ lymphocytes	*R* = −0,6809^*^
MAP	The absolute number of CD3+CCR5+CXCR3+ lymphocytes	*R* = −0,6283^*^
KT/V	The absolute number of CD3+CCR7+ lymphocytes	*R* = 0,6673^*^
KT/V	The absolute number of CD3+CD45RO+CCR7+ lymphocytes	*R* = 0,6832^*^

^*^ *P* < 0.05, Pearson test.
